# High-Power Femtosecond Laser Processing of SiC Ceramics with Optimized Material Removal Rate

**DOI:** 10.3390/mi14101960

**Published:** 2023-10-21

**Authors:** Jian Zhang, Zhichao Liu, Yuanhang Zhang, Feng Geng, Shengfei Wang, Fei Fan, Qinghua Zhang, Qiao Xu

**Affiliations:** Laser Fusion Research Center, China Academy of Engineering Physics, Mianyang 621900, China; zhangjian.leo@163.com (J.Z.); zcliu44@163.com (Z.L.); yuanhang_zhang@126.com (Y.Z.); gengf0326@sina.com (F.G.); robertwsf@sina.com (S.W.); fanfei65790299@163.com (F.F.); zhangqh502@sina.com (Q.Z.)

**Keywords:** femtosecond laser, SiC ceramics, laser processing, material removal rate, surface oxidation

## Abstract

Silicon carbide (SiC) ceramics are widely used as structural materials for various applications. However, the extraordinarily high hardness, brittleness, low material removal rate, and severe tool wear of these materials significantly impact the performance of conventional mechanical processing techniques. In this study, we investigated the influence of different parameters on the material removal rate, surface quality, and surface oxidation during the laser processing of SiC ceramic samples using a high-repetition-frequency femtosecond laser at a wavelength of 1030 nm. Additionally, an experimental investigation was conducted to analyze the effects of a burst mode on the material removal rate. Our results demonstrate that the surface oxidation, which significantly affects the material removal rate, can be effectively reduced by increasing the laser scanning speed and decreasing the laser scanning pitch. The material removal rate and surface quality are mainly affected by laser fluence. The optimal material removal rate is obtained with a laser fluence of 0.4 J/cm^2^ at a pulse width of 470 fs.

## 1. Introduction

Silicon carbide (SiC) ceramics are widely used as structural materials in space optics, aerospace applications, and other industries because of their exceptional thermal conductivity, high hardness, chemical stability, and corrosion resistance [1]. In addition, the fabrication of large-aperture SiC components and complex-shaped SiC products can be relatively easily achieved through sintering [2,3]. Net-shape forming technology, joining techniques [4], and other sintering technologies [5,6] can be employed to meet the shape and structure requirements for manufacturing SiC components. Machining is an indispensable step of the fabrication process, ensuring that the SiC components meet the assembly and functionality requirements [7]. However, conventional mechanical processing methods such as grinding, milling, and drilling encounter challenges when applied to SiC ceramics due to their extraordinary hardness and brittleness. Additionally, cracking and mechanical stress may occur in the processing region owing to the cutting force. Small cracks and tearing defects in SiC ceramics propagate easily, particularly in complex usage scenarios, resulting in disastrous accidents [8].

In recent years, lasers have been used extensively for material processing [9,10]. Ultrafast laser processing is an exceptionally versatile option for many applications that can guarantee a high level of control of the process owing to its ultrashort timescale and ultrahigh peak power density characteristics [11,12]. This technique has already been employed as part of highly selective processing technologies with limited heat-affected zones to provide low distortion, high quality, and precision, particularly for hard and brittle materials [13,14]. Recently developed industrial femtosecond laser sources exhibit high average power (>100 W) and pulse energy (>100 µJ) with a high repetition rate (up to 10 MHz) and short pulse duration (<500 fs) [15,16,17]. By combining the high speed and precision of scanning galvanometers with the properties of high-power femtosecond lasers, these lasers can be applied not only to micro–nano fabrication but also to flexible production processes at high processing speeds and material removal rates with a low total cost of ownership. Furthermore, as high-power femtosecond lasers have become more reliable and efficient, they can be used for automatic processing to meet the demands of flexible production processes [18], particularly for the manufacturing of products with a high demand for yield and cost-effective production. Lastly, ultrafast laser processing is a stress-free method suitable for processing thin-walled components.

Current research on the femtosecond laser processing of SiC and other ceramics has focused on the existing limitations, such as surface oxidation, the processing of fine structures (e.g., micro-holes, micro-grooves), and material thermal damage [18,19,20,21,22,23]. Most of the available studies address parameter optimization, processing path motion strategy, beam shaping, and the effects of assist gas [24,25,26]. However, as large-aperture components or products require significant material removal, achieving a high material removal rate remains a critical factor.

Recent studies have indicated that heat accumulation and particle shielding have a strong impact on the material removal rate and roughness [27,28,29]. However, a comprehensive understanding of the relationship between material removal rate, surface quality, and femtosecond laser parameters for SiC ceramic processing is still lacking. Further investigation is required to explore improvement methods and identify limiting factors for achieving high material removal rates. Furthermore, the laser burst mode for material removal has attracted extensive attention in recent years. Therefore, it is necessary to give a comparative study of conventional single-pulse laser processing and the burst mode strategy [30,31,32]. Consequently, we believe that it is necessary to further study the effects of femtosecond laser fluence on the material removal rate and surface quality of SiC ceramics. 

In this study, we analyzed the effects of different parameters on the material removal rate and surface quality of SiC ceramic samples. We investigated surface oxidation during laser scanning by considering experimental results. Our results show that, with optimized parameters, the material removal rate of the SiC ceramic is directly related to the laser fluence.

## 2. Experimental Setup and Methodology

**Figure 1:**
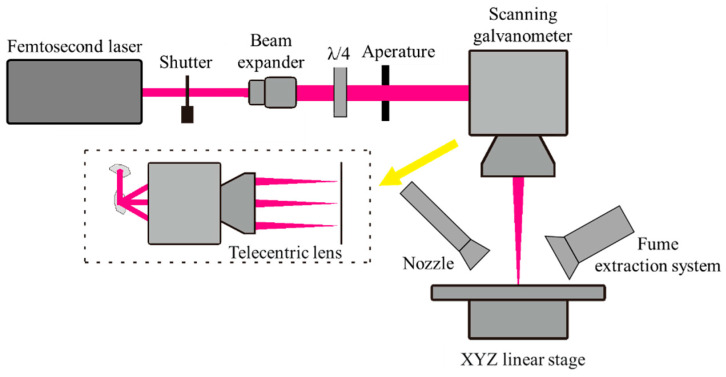
Schematic of the femtosecond laser processing device.

SiC ceramic samples with polished surfaces were utilized in this study. These samples were obtained through vacuum pressureless sintering at a density of 3.10 g/cm^3^. A femtosecond fiber laser (FemtoYL-20, YSL Photonics, Ltd., Wuhan, China) was employed for SiC ceramic processing with the specifications listed in Table 1. A schematic of the device is shown in Figure 1. The parameters of the SiC ceramic samples and laser processing are listed in Table 2.

In our experimental setup, the laser beam is expanded by a 3× beam expander and passes through an aperture to achieve a beam diameter of 8 mm, ensuring the generation of a standard Gaussian beam profile. Then, the laser propagates through a λ/4 plate, providing a circular polarization, which removes the influence of polarization depending on the scanning direction from the material removal rate. Finally, the laser is guided through a scanning galvanometer, delivering a scanning speed of up to 2000 mm/s. We used a telecentric f-theta scanning lens to focus the beam normal to the surface of the entire scanning field. This ensured uniform processing parameters throughout the processing area. The focal depth of the f-theta lenses was 0.6 mm to ensure a uniform laser irradiation dose throughout the depth of the processing area during the experiments. A nozzle was placed near the processing area to provide air sweeps, which can help cool the processing area and reduce the build-up of laser processing products. The air sweep pressure was approximately 0.1 MPa. A fume extraction system was placed opposite the nozzle to collect smoke and dust. The laser power was measured behind the f-theta lens using a laser power meter. All experiments were performed in air at approximately 22 °C.

## 3. Results and Discussions

### 3.1. Morphological Analysis of the Grooves Processed by Femtosecond Laser

**Figure 2:**
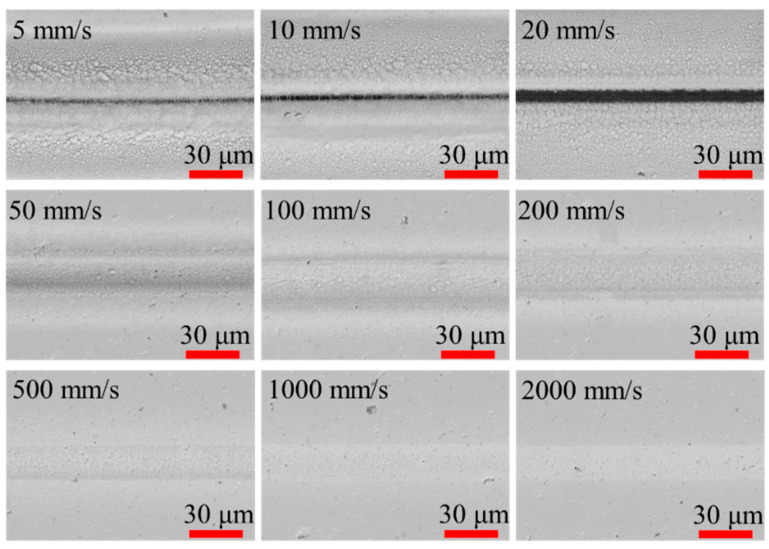
SEM images of grooves processed by the femtosecond laser at different scanning speeds under laser power of 4 W (500 kHz).

**Figure 3:**
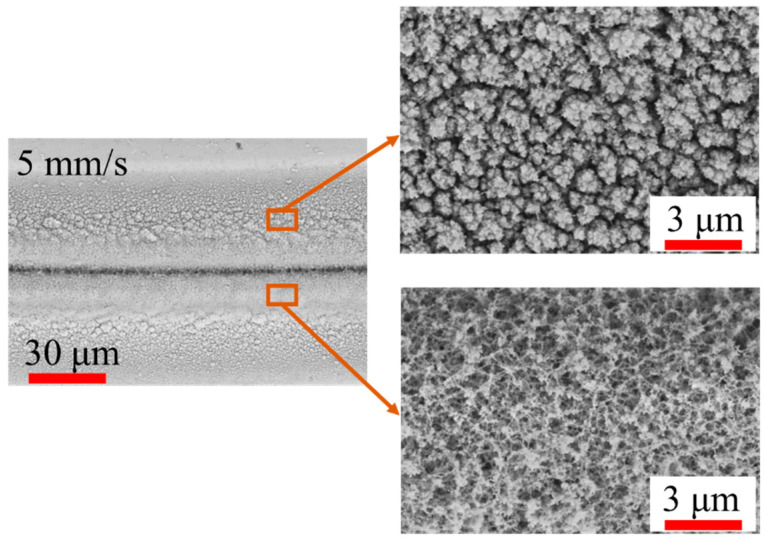
SEM images of produced SiO_2_ at 5 mm/s laser scanning speed.

In this study, a series of experiments were conducted using femtosecond laser processing to analyze the influence of the laser scanning speed on the material removal rate. Analyzing the morphology of the grooves processed by the femtosecond laser under different scanning speeds represents a basic experiment to investigate the thermal effects and changes in the chemical composition of the sample. These experiments can also be used to determine the optimal processing speed. We analyzed the morphologies of laser-processed grooves under different scanning speeds at a laser power of 4 W (500 kHz) using scanning electronic microscopy (SEM, ZEISS Gemini 300, Oberkochen, Germany). These results presented in Figure 2 show that a significant amount of the laser ablation product was deposited on or near the processing grooves when the scanning speed was below 200 mm/s. SiO_2_ is produced when the silicon from the SiC ceramic reacts with oxygen at high temperatures induced by femtosecond laser pulses [18,19,33]. When the scanning speed is low, the SiC ceramic receives more laser energy per unit area, leading to significant heat accumulation. The produced SiO_2_ deposits on the surface, thus preventing the removal of the SiC ceramic. The most abundant SiO_2_ deposits were found at the processing edge and decreased with increasing distance from the processing groove. As shown in Figure 3, the SiO_2_ produced at a laser scanning speed of 5 mm/s presented a white or grey batt-like aspect and had a certain binding force with the surface. By increasing the scanning speed, the amount of laser ablation products gradually decreased. When the scanning speed exceeded 500 mm/s, no obvious oxide deposition was observed on the processed grooves. Therefore, at a fixed power, the amount of oxidation products in the processing area is inversely proportional to the laser scanning speed.

**Figure 4:**
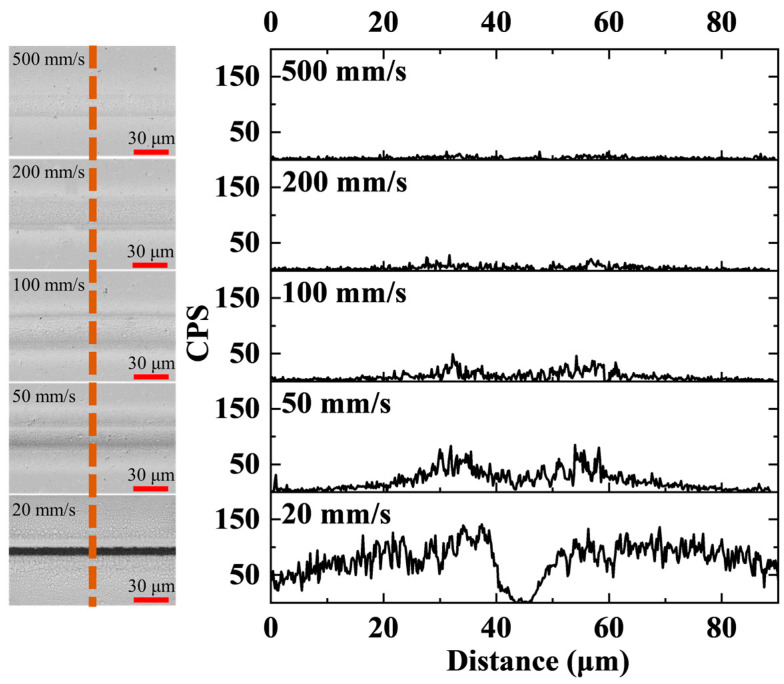
EDS analysis of processing areas at different scanning speeds and 4 W (500 kHz) laser power.

To further investigate the relationship between the scanning speed and oxidation products, we employed energy-dispersive spectroscopy (EDS, ZEISS SmartEDX, Oberkochen, Germany) to analyze the elemental composition of the processing area. The oxygen counts per second (CPS) are shown in Figure 4. These results are consistent with those presented in Figure 2 and Figure 3. In fact, oxygen was most abundant at the edges of the processing grooves and decreased gradually with distance. Notably, when the laser scanning speed exceeded 500 mm/s, the CPS of oxygen were approximately zero, thus indicating that the temperature of the processing area was lower than the oxidation temperature. Our results show that the scanning speed is an important parameter for determining the heat accumulation in the processing area under fixed laser power.

### 3.2. Influence of the Laser Scanning Pitch on Material Removal Rate and Roughness

**Figure 5:**
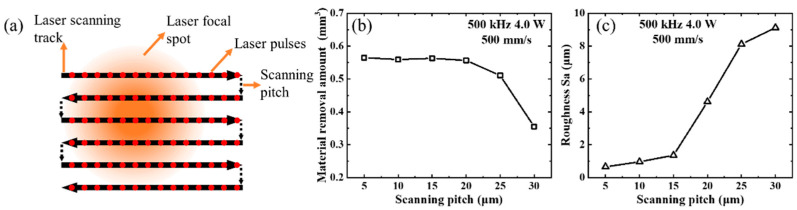
(**a**) Schematic of the laser scanning method. (**b**) Material removal amount and (**c**) roughness of the bottom surface at different laser scanning pitches.

**Figure 6:**
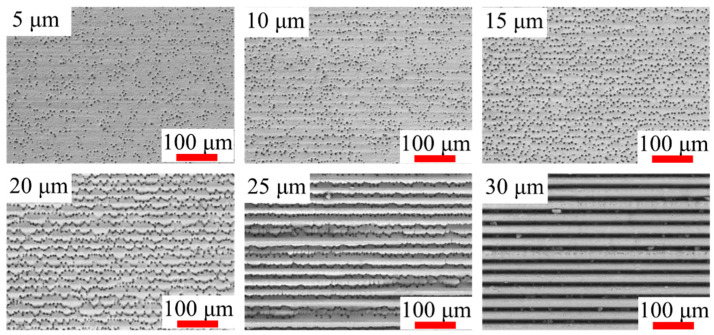
Morphology of the area processed by femtosecond laser at different scanning pitches in the range 5–30 μm under a laser power of 4 W (500 kHz).

The scanning pitch is a crucial parameter that influences the processing morphology and material removal rate. Considering the focal spot size of approximately 36 μm, the scanning pitch was set within the range 5–30 μm. A schematic of the laser-scanning method is shown in Figure 5a. All the experiments in this section were conducted at a laser power of 4 W (500 kHz) and scanning speed of 500 mm/s. The processing area was 1 mm × 20 mm, and each area was processed for 80 s. After processing was completed, the SiC ceramic sample was rinsed by ultrasonic cleaning and measured by laser confocal microscopy to capture the removal depth and measure the surface roughness. The amount of material removed at different scanning pitches is shown in Figure 5b. When the laser scanning pitch was below 20 μm, the amount of material removed was nearly constant, indicating that the thermal effect at different scanning pitches has a limited impact on the material removal rate. However, when the laser scanning pitch was above 20 μm, the amount of material removed decreased sharply, because some of the material at the bottom was not effectively removed. The roughness of the processing area measured through laser confocal microscopy is depicted in Figure 5c. The SEM images of the bottom morphology at different laser scanning pitches are shown in Figure 6. The roughness data in Figure 6 were obtained through laser confocal microscopy. When the laser scanning pitch was below 15 μm, the bottom surface was flat and smooth. However, for scanning pitches greater than 20 μm, an irregular and gully-shaped processed bottom surface emerged. Additionally, the roughness sharply increased to approximately 8 μm, indicating an ineffective removal of material from the bottom surface. These results can be understood considering that the scanning pitch should be at least less than half of the focal spot size because the focal spot has a Gaussian distribution. Considering that the roughness reaches the optimal value of approximately 0.6 μm at a scanning pitch of 5 μm, this value was adopted in this study.

### 3.3. Influence of Laser Scanning Speed and Power on Material Removal Rate and Roughness

**Figure 7:**
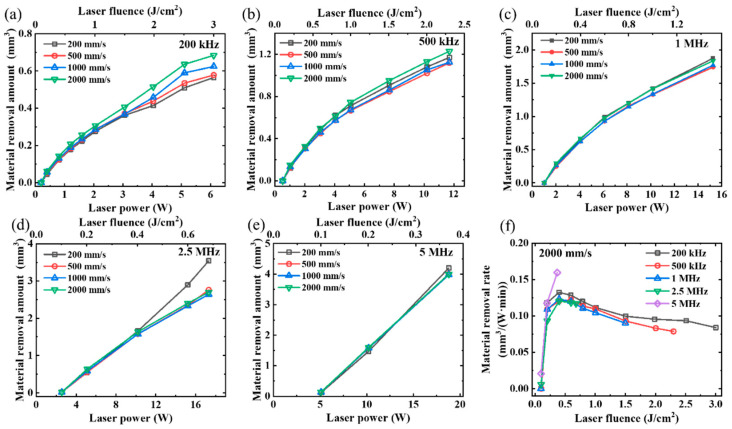
Material removal amount at different laser scanning speeds and powers. Laser repetition frequencies: (**a**) 200 kHz, (**b**) 500 kHz, (**c**) 1 MHz, (**d**) 2.5 MHz, and (**e**) 5 MHz. (**f**) Material removal rate at different laser fluences and repetition frequencies.

In this section, we analyzed the material removal rate under different laser powers, repetition frequencies (200 kHz–5 MHz), and laser scanning speeds (200–2000 mm/s), while maintaining a fixed scanning pitch of 5 μm. As the focal spot (~36 μm) remained constant throughout the experiment, variations in the laser power and repetition rate led to changes in the laser fluence value. All the experiments were performed using a processing area of 1 mm × 20 mm and a consistent processing time of 80 s. The scanning was repeated 4, 10, 20, and 50 times at a scanning speed of 200, 500, 1000, and 2000 mm/s, respectively. The experimental results are presented in Figure 7. The material removal amount exhibited a nonlinear increase with the increment of laser power in Figure 7a–c. The material removal amount during high-speed scanning was slightly higher than that during low-speed scanning. At the laser repetition frequencies of 2.5 and 5 MHz (Figure 7d and Figure 7e, respectively), the material removal amount varied almost linearly with laser power. 

The material removal rates obtained from our experiment are shown in Figure 7f. Although a higher power corresponded to a higher material removal amount, the results in Figure 7f show that the highest material removal rate was achieved at a laser fluence of approximately 0.4 J/cm^2^, regardless of the laser power. However, at this value of laser fluence, the material removal rate at 5 MHz was higher than that at other frequencies, which may be attributed to a decrease in the material removal threshold caused by the rise in the temperature of the substrate. As mentioned in previous studies [30,34], an increase in the temperature of the sample surface reduces the material removal threshold at high repetition frequencies and spot overlap ratios, thus resulting in a higher material removal rate. The same phenomenon happened in Figure 7d. The material removal amount was slightly higher at a scanning speed of 200 mm/s than at higher scanning speeds. It also explains the nonlinear and linear trend of the material removal amount at different laser powers in Figure 7a–e. It should be noted that this linear trend in Figure 7e is merely coincidental and not indicative of a general pattern. An increase in power (>18.8 W) may lead to a more significant heat accumulation effect at 5 MHz, resulting in notable surface oxidation and subsequently reducing the material removal rate.

**Figure 8:**
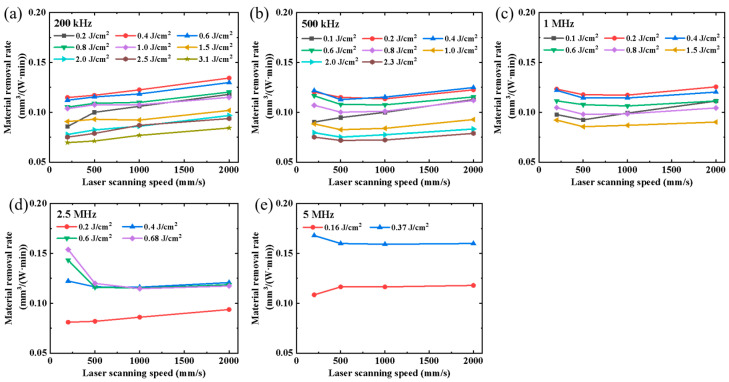
Material removal rate at different laser scanning speeds. Laser repetition frequencies: (**a**) 200 kHz, (**b**) 500 kHz, (**c**) 1 MHz, (**d**) 2.5 MHz, and (**e**) 5 MHz.

The material removal rates at different scanning speeds are illustrated in Figure 8. The highest material removal rate was achieved at a laser fluence of approximately 0.4 J/cm^2^, as depicted in Figure 8, which aligns with the findings presented in Figure 7. However, it should be noted that in Figure 8b–e, the material removal rate at a scanning speed of 200 mm/s surpasses that at other laser scanning speeds, potentially due to a decrease in the material removal threshold caused by an increase in the substrate temperature. This phenomenon was not evident in Figure 8a due to a lower laser repetition frequency. In general, apart from the scanning speed of 200 mm/s, higher scanning speeds can achieve higher material removal rates at all laser fluences.

In conclusion, the optimal material removal amount can be achieved by increasing the laser frequency while keeping the laser fluence unchanged. The optimal laser fluence for the SiC sample used in this study was approximately 0.4 J/cm^2^ at a laser pulse width of 470 fs.

**Figure 9:**
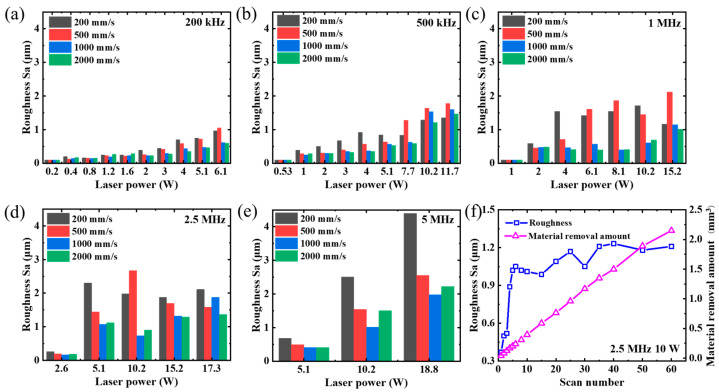
Roughness values of SiC ceramic samples at different laser powers and the following repetition frequencies: (**a**) 100 kHz, (**b**) 500 kHz, (**c**) 1 MHz, (**d**) 2.5 MHz, and (**e**) 5 MHz. (**f**) Roughness values at different scanning numbers, a laser repetition frequency of 2.5 MHz, a laser power of 10.2 W, a laser fluence of 0.4 J/cm^2^, and a scanning speed of 2000 mm/s.

Due to the limited number of studies investigating the factors influencing the roughness of SiC ceramics, we analyzed this feature by varying the laser fluence and pulse repetition. The surface roughness was measured using laser confocal microscopy. The relationship between the laser power, repetition frequency, and roughness is shown in Figure 9. The roughness depended significantly on the laser fluence and scanning speed. Specifically, it increases with higher laser powers at a fixed frequency. At a scanning speed of 2000 mm/s and laser power below 5 W, the roughness ranged between 0.5 and 1.1 μm, regardless of the frequency. However, when increasing the laser power above 10 W, the roughness ranged from 0.7 to 2.2 μm. At scanning speeds of 200 and 500 mm/s, the roughness was higher than that at 1000 and 2000 mm/s at the same power, possibly owing to heat accumulation effects. These results suggest that a scanning speed exceeding 1000 mm/s represents an optimal choice within our investigated range of laser powers for achieving a smooth sample surface. 

The roughness values of the bottom surface and the material removal at different scanning numbers are depicted in Figure 9f. The laser repetition frequency, power, fluence, and scanning speed were set to 2.5 MHz, 10 W, 0.4 J/cm^2^, and 2000 mm/s, respectively. The surface roughness initially increased with increasing scanning numbers and then remained nearly constant at a value of 1.2 μm.

### 3.4. Influence of the Laser Burst Mode on Material Removal Rate

**Figure 10:**
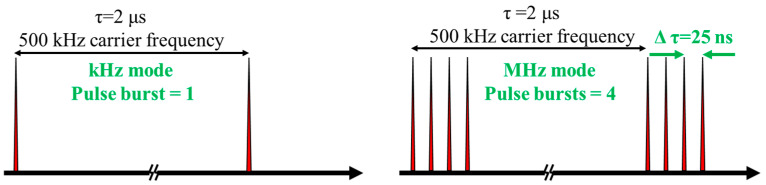
Examples of laser-generated burst modes.

**Figure 11:**
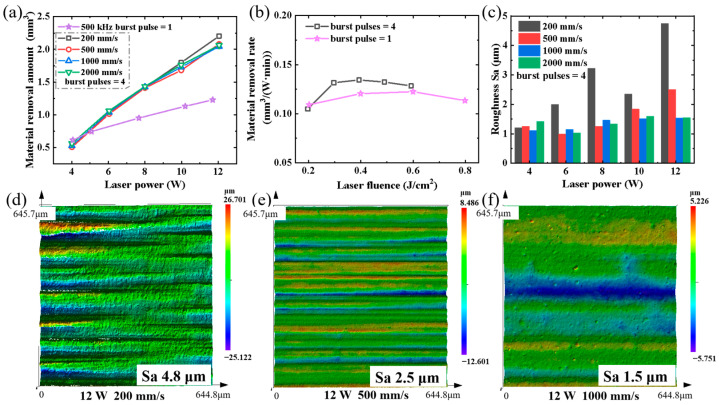
(**a**) Material removal amount, (**b**) material removal rate, and (**c**) roughness at different values of laser power and scanning speed. Surface roughness micrographs at scanning speed of (**d**) 200 mm/s, (**e**) 500 mm/s, and (**f**) 1000 mm/s under laser power of 12 W.

In the past decade, femtosecond laser processing in burst mode has gained increasing attention [32]. Burst modes consisting of multiple pulses with a delay of several 10 ns enable innovative processing regimes, leading to improved material removal rates and surface quality [33]. However, several studies have also shown that an increase in the pulse repetition rate may reduce the ablation efficiency and surface quality due to plasma and particle shielding, as well as heat accumulation [34]. Additionally, when processing SiC ceramics in air, heat accumulation may also lead to oxide deposition in the processing area, thus affecting the material removal rate. Therefore, we developed an experimental setup to investigate the burst mode associated with heat accumulation and its impact on surface quality. Figure 10 illustrates a burst mode where pulses were generated by the laser oscillator every 25 ns (40 MHz) and grouped into identical trains at 2 μs intervals. Our experiments were conducted at various scanning speeds and laser powers with a pulse repetition rate of 500 kHz and a train of four burst pulses. The processing area was 1 mm × 20 mm, and each area was processed for 80 s. As shown in Figure 11, when the laser power was above 6 W, the material removal amount in burst mode increased significantly compared to that in the traditional laser mode. For example, at a laser power of 12 W (Figure 11a), the material removal amount in burst mode reached approximately 2.1 mm^3^, while it was only around 1.22 mm^3^ without utilizing burst mode. However, considering values of laser fluence in the range of 0.3–0.5 J/cm^2^, the highest material removal rate in burst mode was only approximately 10% higher than that in traditional mode (Figure 11b). These results indicate that the material removal rate is mainly affected by the laser fluence.

We further investigated the impact of the burst mode on surface roughness by varying laser power and scanning speed. As shown in Figure 11c, the roughness in burst mode is significantly influenced by the scanning speed. Under an identical laser power, at a scanning speed of 200 mm/s, the roughness ranged between 1.2 and 4.5 μm, whereas at a scanning speed of 2000 mm/s, its values were in the range 1–1.6 μm. These results are consistent with those obtained in the traditional laser mode presented in Figure 9. However, owing to the shorter pulse interval of the burst mode, heat accumulation and particle shielding had a more pronounced influence on the surface quality, leading to higher roughness values compared to those obtained with the traditional mode. As shown in Figure 11d–f, the micrographs described the surface roughness of the processing area at scanning speeds of 200 mm/s, 500 mm/s, and 1000 mm/s under a laser power of 12 W.

### 3.5. Thermal Infrared Images of the Processing Areas

**Figure 12:**
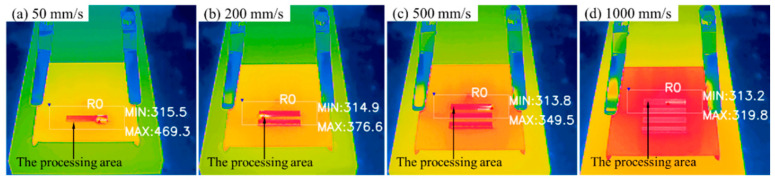
Thermal infrared images of processing areas at different scanning speeds: (**a**) 50 mm/s, (**b**) 200 mm/s, (**c**) 500 mm/s and (**d**) 1000 mm/s (temperature is indicated by Kelvin (k)).

We used an infrared camera with a measurement range of 273–469 K to measure the temperature of the processing areas at different values of laser power and scanning speed with a laser repetition frequency of 2.5 MHz (10.2 W, 0.4 J/cm^2^). Each processing area of 3 mm × 20 mm was processed for 10 min; photographs were taken after 9 min. The thermal infrared images presented in Figure 12 show that the heat accumulation effect became more evident as the scanning speed decreased. At a scanning speed of 50 mm/s, the maximum surface temperature exceeded 469 K. Conversely, when the scanning speed exceeded 500 mm/s, no significant increase in temperature was observed in the processing area compared to the ambient temperature after a processing time of 9 min. These results demonstrate that optimized parameters can achieve high-efficiency processing of SiC ceramics without significant heat accumulation.

## 4. Conclusions

In this study, the effects of high-power, high-repetition-frequency femtosecond laser irradiation on SiC ceramics were experimentally investigated. We systematically analyzed the effects of different parameters on the material removal rate, surface quality, and oxidation during laser processing. Our results showed that increasing the laser scanning speed and decreasing the laser scanning pitch are effective methods to reduce surface oxidation. Additionally, both the material removal rate and surface quality are mainly affected by the laser fluence. In particular, the optimal material removal rate was achieved at a laser fluence of 0.4 J/cm^2^. Lastly, our experiments confirmed that the burst mode can be used to moderately improve the material removal rate by approximately 10% at the optimal laser fluence. To investigate the impact of heat accumulation at different scanning speeds on surface oxidation, we used thermal infrared images to analyze the impact of heat accumulation at different scanning speeds. Our results showed that no significant temperature increase was observed in the processing area at the optimized parameters. In summary, this study proves that optimized parameters can achieve high-efficiency processing of SiC ceramics without significant heat accumulation.

## Figures and Tables

**Table 1:** Specifications of the femtosecond fiber laser.

Parameters	Value
Laser wavelength	1030 ± 5 nm
Pulse energy (max)	40 μJ
Pulse width (FWHM)	470 fs
Repetition frequency	50/100/200/300/500/800 kHz, 1/1.2/2.5/5 MHz
Power (max)	20 W
Laser mode	TEM00 (M^2^ < 1.3)

**Table 2:** Specifications of SiC ceramic samples and laser processing parameters.

Parameters	Value
Sample roughness (Sa)	0.1 μm
Sample side length	50 mm
Sample thickness	4 mm
Focal length	100 mm
Focal spot	36 μm
Laser scanning speed	1–2000 mm/s

## Data Availability

Data underlying the results presented in this paper are not publicly available at this time but may be obtained from the authors upon reasonable request.
